# An Unusual and Severe Thyrotoxicosis in a Twin Pregnancy: Fortune Favors the Brave

**DOI:** 10.1155/crie/6298137

**Published:** 2025-01-10

**Authors:** Virginia Maltese, Elisa Gatta, Irene Silvestrini, Valentina Anelli, Francesca Bambini, Caterina Buoso, Maria Cavadini, Massimiliano Ugoccioni, Maura Saullo, Fiorella Marini, Elena Gandossi, Andrea Delbarba, Ilenia Pirola, Carlo Cappelli

**Affiliations:** ^1^Department of Clinical and Experimental Sciences, University of Brescia, Brescia, Italy; ^2^SSD Endocrinologia, ASST Spedali Civili, Brescia, Italy; ^3^Centro per la Diagnosi e Cura delle Neoplasie Endocrine e delle Malattie della Tiroide, University of Brescia, Brescia, Italy; ^4^Department of Internal Medicine and Therapeutics, University of Pavia, Pavia, Italy

## Abstract

Graves' disease (GD) and gestational transient thyrotoxicosis (GTT) are the most common causes of thyrotoxicosis during pregnancy, with prevalence ranging from 0.1% to 1% and from 1% to 3%, respectively. Hyperthyroidism during pregnancy can have severe consequences if not promptly recognized and treated. Even more severe, if possible, is the thyroid storm, a life-threatening complication of hyperthyroidism, characterized by severe and dramatic clinical manifestations of thyrotoxicosis. No prior history of thyroid disease, absence of GD stigmata, negative thyrotropin (TSH) receptor antibody levels, serum human chorionic gonadotropin (hCG) higher on average, and symptoms of emesis may lead to the diagnosis of GTT. Few cases of thyroid storm during pregnancy are reported in literature, mainly due to gestational trophoblastic disease. We report a rare and severe case of thyroid storm in a 24-year-old woman at 15 weeks' gestation with twins, likely due to GTT, precipitated by acute myocarditis. Initially presenting with weakness, vomiting, and sinus tachycardia, the patient rapidly deteriorated into a life-threatening condition characterized by hypokalemia, myocardial injury, and severe thyrotoxicosis. Cardiac imaging later revealed acute myocarditis. Thyroid function stabilized at the end of the pregnancy, allowing discontinuation of methimazole. Both fetuses were delivered via emergency cesarean section at 36 weeks, with no significant congenital abnormalities. This case highlights the complexity of diagnosing and managing hyperthyroidism in twin pregnancies, particularly in the context of hyperemesis gravidarum (HG).

## 1. Introduction

Approximately 3% of live newborns are from twin pregnancies [1], characterized by higher risk for some adverse outcome compared to singleton ones, both maternal and fetal [2]. The latter includes preterm birth, low birth weight, intrauterine growth restriction (IUGR), and twin-to-twin transfusion syndrome in monochorionic pregnancies [3, 4]. Most frequent maternal complications are represented by gestational hypertension and preeclampsia, gestational diabetes, intrahepatic cholestasis, and hyperemesis gravidarum (HG) [2, 5–7].

Graves' disease (GD) is the leading cause of hyperthyroidism, occurring 5–10 times more frequently in women, primarily of childbearing age, in developed countries [8]. GD and gestational transient thyrotoxicosis (GTT) are the most common causes of thyrotoxicosis during pregnancy, with prevalence ranging from 0.1% to 1% and from 1% to 3%, respectively [9].

Hyperthyroidism during pregnancy can have severe consequences if not promptly recognized and treated [10]. Maternal complications are represented by preeclampsia and gestational heart failure [11, 12], whereas fetal complications can include low birth weight, premature labor, fetal hyperthyroidism, and increased neonatal and perinatal mortality [12–14]. Even more severe, if possible, is the thyroid storm, a life-threatening complication of hyperthyroidism, characterized by severe and dramatic clinical manifestations of thyrotoxicosis [15].

In the absence of specific findings of GD, such as goiter or orbitopathy, the differential diagnosis between GD and GTT could be challenging, even if mandatory. In fact, antithyroid drugs (ATDs) are not recommended for GTT [16], both due to its almost always self-limiting nature and the well-known maternal–fetal toxic effects of ATDs [10, 16–19]. No prior history of thyroid disease, absence of GD stigmata, negative thyrotropin (TSH) receptor antibody (TRAb) levels, serum human chorionic gonadotropin (hCG) higher on average, and symptoms of emesis may lead to the diagnosis of GTT [16]. However, the clinical presentations can sometimes be inconclusive.

Few cases of thyroid storm during pregnancy are reported in literature, mainly due to gestational trophoblastic disease [17, 20–28]. We report an unusual and severe case of thyroid storm in a healthy young woman pregnant with twins, probably due to GTT, in accordance with the Case Report (CARE) checklist [29].

## 2. Case Presentation

A 24-year-old woman at 15 weeks with twin gestation (gravida 2, para 1) was referred to the obstetrics emergency department on November 16, 2023, due to weakness, repeated vomiting, and an inability to eat or drink. She had been healthy prior and was not on any chronic medications. Since the beginning of pregnancy, she had been taking doxylamine/pyridoxine and metoclopramide for nausea and vomiting. In September 2023, routine blood exams were normal, and thyroid function tests showed a TSH level of 0.87 mIU/L (normal range 0.27–4.20).

Physical examination showed mild agitation, tachycardia (120 beats per minute [bpm]), fever (37.5°C), and no tremors. Arterial blood pressure was 115/80 mmHg, and oxygen saturation was 97%.

**Figure 1:**
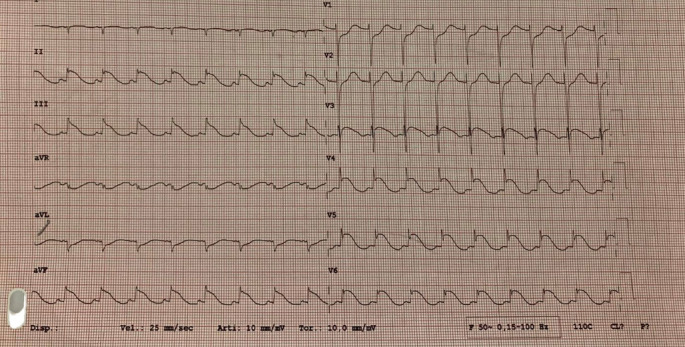
Electrocardiogram (ECG) at the admission.

An electrocardiogram (ECG) revealed sinus tachycardia (104 bpm), ST-segment elevation in the inferolateral region, and a prolonged QTc interval (603 ms) (Figure 1). Laboratory tests revealed severe hypokalemia (K^+^ 1.9 mmol/L, normal range 3.4–4.5) and hyponatremia (Na^+^ 118 mmol/L, normal range 136–145) which were promptly identified and adequately corrected.

Cardiac enzymes were elevated (troponin T 9082 ng/L [normal range <14], creatine kinase MB 420 mcg/L [normal range <7]), and a transthoracic color Doppler echocardiogram (TTE) showed inferior cardiac hypokinesia with an ejection fraction (EF) of 55%. After obstetrical consultation cardiac, magnetic resonance imaging (MRI) was not performed as at any time during pregnancy could be associated with an increased risk of a broad set of rheumatological, inflammatory, or infiltrative skin conditions and for stillbirth or neonatal death [30].

On November 17, the patient's clinical condition rapidly deteriorated, necessitating a transfer to the intensive care unit for a sustained episode of ventricular tachycardia with loss of pulse, requiring an electric shock to restore sinus rhythm, although sinus tachycardia persisted. Coronary angiography revealed normal coronary arteries, and an intra-aortic balloon pump (IABP) was inserted to support hemodynamic function impaired from refractory arrythmia; a follow-up TTE showed a further worsening of the EF to 40%. Concurrently, respiratory function deteriorated for the presence of massive bilateral pleural effusion, leading to the need for noninvasive mechanical ventilation followed by orotracheal intubation and invasive mechanical ventilation. Empirical antibiotic and steroid therapy were stared in the suspicious of myocarditis.

Blood cultures, cardiotropic virus screening, autoimmunity tests, and thyroid function tests were performed. All tests were negative except for significant thyrotoxicosis (TSH <0.005 mIU/L, free thyroxine [fT4] >77 ng/L [normal range 9.3–17.0]). TRAb, antithyroid peroxidase, and antithyroglobulin antibodies were negative; hCG was two times upper the reference range for gestational age (121,036 mIU/mL, normal range for gestational age 13,950–56,451). Biochemical data are summarized in Table 1.

At the endocrinological evaluation, no goiter or exophthalmos were evidenced, and the Burch–Wartofsky score was 45 (body temperature 37.5°C, heart rate 135 bpm, moderate congestive heart failure, and moderate gastrointestinal and hepatic disturbances). Ultrasound showed a mild increase goiter, with usual echogenicity and no nodule. A diagnosis of thyroid storm probably due to gestational thyrotoxicosis was made, and high-dose methimazole therapy (10 mg every 8 h) was quickly initiated, along with beta-blockers and steroids.

**Figure 2:**
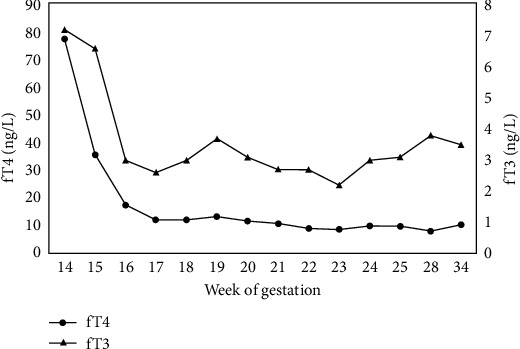
Trend of serum thyroxine (fT4) and triiodothyronine (fT3) during pregnancy.

Following the initiation of antithyroid therapy, the patient's condition improved rapidly concurrently with a fT3 level decrease until normalization within 10 days. Clinically, EF improved to 50%, she was extubated after 5 days, and the IABP was removed after 6 days. Thyroid function tests showed progressive improvement by the 18th week of gestation, allowing the suspension of steroid therapy first, followed by the gradual reduction of methimazole (Figure 2).

After 3 months of hospitalization, the patient was discharged and was followed up in the endocrinology outpatient clinic with monthly monitoring of thyroid function, which improved, allowing for a reduction and discontinuation of methimazole at 36 weeks of gestation.

During her hospitalization, regular and periodic obstetric–gynecological monitoring was performed. IUGR of both fetuses was observed, and amniocentesis revealed a 46 XX karyotype with no abnormalities.

In April 2024, at 36 weeks of gestation, the patient underwent an emergency cesarean section due to cardiotocographic abnormalities. Two baby girls were born, both small for gestational age (SGA) and apparently normoconformed. Neuropsychiatric evaluation, metabolic tests (including TSH measurement), congenital cataract screening, and audiological screening were all normal. The first baby developed transient neonatal tachypnea at birth, requiring noninvasive respiratory support, and was admitted to the neonatal intensive care unit. The second baby developed necrotizing enterocolitis at 15 days of life, which was successfully treated with oxygen, antibiotics, and supportive therapy. Subsequent follow-up for both babies after discharge was normal.

Following childbirth, the patient remained in good health. Repeated outpatient thyroid function testing yielded a normal result in subsequent months, and she had no recurrence of symptoms. She underwent cardiac MRI, revealing findings suggestive of a recent myocarditic and a phenotype of nondilated left ventricular cardiomyopathy (NDLVC), associated with extensive nonischemic tissue alterations. In light of this, the patient was deemed eligible for implantation of a cardioverter-defibrillator as a secondary prevention.

## 3. Discussion

GTT is typically a temporary complication occurring in the first trimester of pregnancy, characterized by elevated thyroid hormone levels (hyperthyroxinemia) due to overstimulation of the TSH receptor by the alpha subunit of hCG, in a manner similar to that of TSH. GTT affects up to 60% of pregnancies complicated by HG, which involves severe nausea and vomiting in early pregnancy, resulting in more than 5% weight loss, dehydration, and ketonuria [10, 16]. GTT is typically transient and resolves spontaneously as hCG levels decrease after peaking between 8 and 15 weeks of gestation [10, 19, 31]. Women with twin pregnancies, a common cause of GTT, have elevated hCG levels for a longer duration, which may result in more pronounced symptoms [32].

Since GTT is transient and ATDs could be teratogenic during the first trimester, unlike in GD, treatment with ATDs is generally not recommended. Instead, supportive care, including hydration, antiemetics, and electrolyte replacement, is advised [10, 16–18]. According to the literature, ATDs should be reserved for patients with severe GTT, where the risk posed by overt hyperthyroidism to both the mother and fetus outweighs the potential harm of fetal exposure to ATDs [16, 19].

Thyroid storm is a rare complication of hyperthyroidism with a mortality rate up to 30% if not recognized immediately and aggressively treated [24, 33]. Thyroid storm during pregnancy is a very rare condition, and its incidence remains unclear [34]. A population-based retrospective cohort study of a Canadian database reported its prevalence in 2.97 per 1,000,000 pregnancies [35].

The principles of treatment are based on clinical experience, as there are no prospective studies, particularly in pregnant women. Identifying and addressing any precipitating factors (e.g., infection), along with specific therapy targeting the thyroid, may be crucial to the outcome. Comprehensive patient support in an intensive care unit is essential [33, 36, 37]. The therapeutic options are expanded from those used for uncomplicated hyperthyroidism given in higher doses (thionamides, methimazole or propylthiouracil, and beta-blockers), in addition to glucocorticoids [38, 39].

Theoretically, propylthiouracil might initially be preferred because of extrathyroidal inhibition of thyroxine conversion to triiodothyronine in addition to its inhibitory effect on thyroid hormones synthesis. On the other hand, methimazole, because of its long serum half-life and slow intrathyroidal turnover, as because methimazole is not protein-bound, may be favored thanks to its longer duration of action compared to propylthiouracil [40]. In addition, a recent meta-analysis by Isozaki et al. [41] compared the efficacy and safety of propylthiouracil and methimazole reporting that methimazole was superior to propylthiouracil as regards reduction of thyroid hormones levels, minimal risk of liver function damage, and increase in TSH levels. For these reasons and in accordance with current Guidelines of the American Thyroid Association for the Diagnosis and Management of Thyroid Disease During Pregnancy [16], the patient was treated with methimazole.

To the best of our knowledge, in nearly all cases of thyrotoxic crisis in women with HG, methimazole or propylthiouracil has been used, leading to rapid control of thyroid function until discontinuation around the 18th to 20th week of gestation, due to the physiological decrease in hCG levels [17, 20, 23, 42–44]. Unlike typical cases, our patient did not experience remission upon entering the second trimester, requiring discontinuation of ATDs only at the end of the pregnancy. This significant detail made us question our diagnosis, despite the absence of pathognomonic signs of GD. A similar case was reported by Coulon et al., describing a mutation (Lys183Asn) in the TSH receptor gene [43], which was associated with increased sensitivity to hCG and a prolonged period of thyrotoxicosis [45]. Unfortunately, we did not perform genetic sequencing, which prevents us from drawing any conclusions on this matter. However, we emphasize that the patient did not experience GTT during her previous pregnancy, making a mutation highly unlikely.

Although thyroid storm can occur in patients with longstanding untreated hyperthyroidism, it is often precipitated by an acute event such as thyroid or nonthyroidal surgery, trauma, infection, or an acute iodine load. Additionally, irregular use or discontinuation of ATDs and limited access to healthcare are commonly reported triggers of thyroid storm [36, 37, 46]. Certainly, our patient did not suffer from longstanding hyperthyroidism, as evidenced by the thyroid function tests from September. It is highly plausible that a precipitating event, such as myocarditis, may have played a key role in the development of the thyrotoxic crisis in the context of HG. In fact, an MRI performed 1 month after delivery revealed recent myocarditis in the context of NDLVC, leading to the recommendation for implantation of a cardioverter-defibrillator as a secondary preventive measure.

It is well-known that hyperthyroidism during pregnancy can lead to various maternal and fetal complications, including adverse neurocognitive outcomes [47, 48]. Moreover, the risk of maternal and fetal adverse effects from ATDs, although it varies across studies, should not be underestimated [48]. ATDs have, in fact, been associated with potential teratogenic effects, particularly when administered between gestational weeks 6 and 10 or earlier [49].

On the other hand, a recent meta-analysis clearly demonstrated that restoring a euthyroid state reduces the incidence of maternal and fetal complications [50]. We chose to treat the patient with methimazole, and as fortune favors the brave, she has given birth to two children, both of whom are currently in excellent health.

In conclusion, our case may represent the first instance of thyroid storm in a woman with twin pregnancy-associated HG, precipitated by acute myocarditis. TSH evaluations throughout pregnancy, especially in twin pregnancies and particularly when signs or symptoms of HG are present, should be suggested.

## Figures and Tables

**Table 1:** Blood exam in acute and recovery phase.

Laboratory tests	Normal value	Acute phase (14 weeks)	Recovery phase (23 weeks)	Delivery (36 weeks)
Red blood cells (×10^6^/µL)	4.0–5.2	4.45	4.09	11.5
White blood cells (×10^3^/µL)	4.0–10.8	16.6	15.1	4.52
Platelets (×10^3^/µL)	130–400	379	284	241
Aspartate aminotransferase (IU/L)	18–34	424	15	16
Alanine aminotransferase (IU/L)	10–35	65	9	13
Gamma-glutamyl transferase (IU/L)	6–42	18	12	4
Bilirubin (mg/dL)	<1.20	1.05	0.21	0.36
Potassium (nmol/L)	3.4–4.5	1.9	3.9	4.2
Sodium (nmol/L)	136–145	118	137	135
Troponin T (ng/L)	<14	9082	8	4.1
Creatine kinase MB (µg/L)	<7	420	2.8	4
Thyroid-stimulating hormone (mIU/L)	0.27–4.20	<0.008	0.014	2.06
Free thyroxine (ng/L)	9.3–17.0	>77	9.1	10.4
Free thyronine (ng/L)	2.2–4.0	NA	2.7	3.5
Ab anti-TSH receptor (IU/L)	<1.8	<1.1	NA	NA
Ab antithyroid peroxidase (kIU/L)	<34	<15	NA	NA
Ab antithyroglobulin (kIU/L)	<15	24.2	NA	NA
Human chorionic gonadotropin (mIU/mL)	13,950–56,451	121,036	NA	NA

## Data Availability

The data that support the findings of this study are available from the corresponding author upon reasonable request.
